# Increased IL17A, IFNG, and FOXP3 Transcripts in Moderate-Severe Psoriasis: A Major Influence Exerted by IL17A in Disease Severity

**DOI:** 10.1155/2016/4395276

**Published:** 2016-11-30

**Authors:** Priscilla Stela Santana de Oliveira, Michelly Cristiny Pereira, Simão Kalebe Silva de Paula, Emerson Vasconcelos Andrade Lima, Mariana Modesto de Andrade Lima, Rodrigo Gomes de Arruda, Wagner Luís Mendes de Oliveira, Ângela Luzia Branco Pinto Duarte, Ivan da Rocha Pitta, Moacyr Jesus Melo Barreto Rêgo, Maira Galdino da Rocha Pitta

**Affiliations:** ^1^Laboratório de Imunomodulação e Novas Abordagens Terapêuticas (LINAT), Núcleo de Pesquisa em Inovação Terapêutica Suely Galdino (NUPIT-SG), Universidade Federal de Pernambuco (UFPE), Recife, PE, Brazil; ^2^Hospital das Clínicas, Universidade Federal de Pernambuco (UFPE), Recife, PE, Brazil; ^3^Faculdade Nova Roma, Recife, PE, Brazil

## Abstract

Psoriasis is a chronic and recurrent dermatitis, mediated by keratinocytes and T cells. Several proinflammatory cytokines contribute to formation and maintenance of psoriatic plaque. The Th1/Th17 pathways and some of IL-1 family members were involved in psoriasis pathogenesis and could contribute to disease activity. Therefore, we sought to analyse skin transcript levels of IL17A, IL22, RORC, IL8, IFNG, IL33, IL36A, FOXP3, and IL10 and correlate with clinic of patients with plaque-type psoriasis. In order to conduct that, we collected punch biopsies from lesional skin and obtained tissue RNA. After reverse transcription, qRT-PCR quantified the relative mRNA expression. The main results revealed increased transcripts levels of IL17A, IFNG, and FOXP3 in moderate-severe patients. Despite this, only IL17A can increase the chance to worsen disease severity. We also observed many significant positive correlations between each transcript. In conclusion, IL17A is elevated in lesional skin from psoriasis patients and plays crucial role in disease severity.

## 1. Introduction

Plaque-type psoriasis is the most prevalent form of psoriasis, corresponding to approximately 90% of cases. Erythematous scaly plaques, well demarcated, raised edges, and varied distribution throughout the body [1] characterize classical lesions. In some cases, systemic diseases such as inflammatory bowel disease and cardiovascular complications present worsening symptoms [2, 3].

The Th1/Th17 pathways are the principal immune components of the disease. The precise mechanism of how the plaque is formed remains uncertain, but when some kind of “triggers” are activated in skin, a cascade of molecules acts in the interaction between keratinocytes and immune cells. At the beginning of the process, interferon gamma (IFN*γ*) and tumor necrosis factor alpha (TNF*α*) induce Langerhans cells to produce IL-12 and IL-23 [4]. IL-12 contributes to the strengthening of Th1 profile while IL-23 leads to Th17 differentiation that produces, mainly, IL-17 and IL-22 [5, 6]. In the epidermis, IL-8 operates as a potent chemotactic factor for neutrophils and contributes to the development of the erythema observed in skin lesions [7].

Recent studies have pointed out the active participation of others cytokines from IL-1 family. Between them there is IL-33, also named IL-1F11, that mediates biological functions through IL-1 orphan receptor ST2 [8]. In psoriasis, TNF*α* regulates IL-33, which promotes inflammation through mast and keratinocyte activation [9, 10]. In addition, IL-36*α* is one of the three homology proteins IL-36*α* (IL-1F6), *β* (IL-1F8), and *γ* (IL-1F9) that also belong to the IL-1 family. They are expressed in both dendritic and keratinocytes cells [11]. Evidence of the involvement of IL-36 cytokines in the pathophysiology of psoriasis includes the fact that a nonfunctional receptor antagonist (IL-36Ra) was associated with generalized pustular psoriasis [12]. Furthermore, IL-36 ligands deficient mice were protected from psoriasis form dermatitis model while the absence of IL-36Ra exacerbated the pathology [13].

On the other hand, regulatory T cells seem to fail in their peripheral anti-inflammatory control. Several researchers found decreased CD4^+^CD25^+^FOXP3^+^ T circulating cells both numerically and functionally in psoriasis patients [14, 15]. In order to balance the deficient anti-inflammatory response, clinical trials have been done using IL-10 recombinant human (rh) therapy protocols [16].

The aim of this study was to evaluate the pro- and anti-inflammatory psoriasis panel of molecules. Here, we sought to quantify Th17-related (IL17A, IL22, and RORC), Th1-related (IFNG and IL8), Treg-related (FOXP3 and IL10), and IL-1 family (IL33 and IL36A) skin transcripts and correlate with disease activity, systemic comorbidities, and methotrexate use in samples of Brazilian patients affected by psoriasis.

## 2. Materials and Methods

### 2.1. Ethics Committee

The human ethics committee of the Health Sciences Center of the Federal University of Pernambuco, located in Recife, Brazil, approved the study protocol (process number: 723.390).

### 2.2. Population Study

The study included twenty-one patients (11 male and 10 female) with plaque-type psoriasis attended and randomly selected in the Dermatology and Rheumatology Outpatient Clinic at Universidade Federal de Pernambuco. Only patients diagnosed with plaque-type psoriasis in strict accordance with the diagnostic criteria of Nestle et al. [17] with no prior immunobiologic therapy and no coexistent autoimmune disorders were considered. Psoriasis Area and Severity Index (PASI) was measured and classified as mild (0–10) and moderate-severe (>10) according to Menter et al. [18]. Other clinical parameters as comorbidities, disease duration, and previous systemic treatment were also questioned.

### 2.3. Skin Samples and RNA Extraction

Four milimeters (4 mm) punch biopsies were taken from lesional skin of psoriatic patients. They were stored up to 24 hours at 4°C RNA later stabilization solution (Invitrogen Life Technologies, CA, EUA) until extraction. RNA was isolated using QIAGEN RNeasy Kit (Qiagen, Valencia, CA) and the amount was measured by nanodrop 2000 (Thermo Fisher Scientific, Carlsbad, CA, EUA). The maximum of 500 ng of total tissue RNA was reverse transcribed using High-capacity cDNA archive Kit 2X (Applied Biosystems Warrington, UK) according to manufacturer's instruction.

### 2.4. Quantitative Real-Time Polymerase Chain Reaction Analysis

qRt-pcr was carried out using predesigned Taqman probes gene expression assay (Applied Biosystems, Warrington, UK), using ABI Prism 7900 HT sequence detection PCR machine (Applied Biosystems, Warrington, UK). We evaluated IL8 (Hs00174103_m1), IFNG (Hs00989291), IL-33 (Hs00369211_m1), IL36A (Hs00205367), IL17A (Hs00174383), IL22 (Hs01574154), IL10 (Hs00961622), RORC (Hs01076122_m1), FOXP3 (Hs01085834_m1), and 18S (Hs03928990) as a housekeeping gene. The cycling condition consisted of 2 minutes at 50°C followed by 10 minutes at 95°C. After these steps, there are 40 cycles at 95°C for 15 seconds and at 60°C for 1 minute.

### 2.5. Statistical Analysis

We used GraphPad PRISM 6.01 software (GraphPad Software Inc., San Diego, CA) and STATA 12 (StataCorp LP., Texas, USA) for data plotting and analysis. To ascertain the sample's normality, we performed D'Agostino & Pearson omnibus normality test. The Mann–Whitney test and Spearman rank correlation were used when the variables did not follow Gaussian distribution. For variables that passed normality test, we applied unpaired *t*-test. We considered correlation (*R* ^2^) strength as follows: 0 < *R* ^2^ ≤ 0.35 = weak correlation; 0.35 < *R* ^2^ ≤ 0.67 = moderate correlation; 0.67 < *R* ^2^ ≤ 1 = strong correlation. To evaluate the association between transcripts levels and PASI, we performed multiple logistic regression for the clinical variables with dichotomous scores. The statistical significance was accepted when *p* < 0.05.

## 3. Results

### 3.1. Patients Cohort

A group of 21 patients, 11 men and 10 women, was included in this study. The mean age was 52 years with men showing a lower mean age than women (46.9 and 57.7, resp.). We stratified the PASI in accordance with the classification postulated by Menter and colleagues (2008), in the Journal of American Academy of Dermatology. PASI showed 8 as the lower punctuation and 28 as the highest score. General mean of PASI was 18 ± 7.2. According to that, men showed more severe disease (mean 21.3 ± 5.8) than women (14.4 ± 7.2) and it was statistically significant, *p* = 0.026. Thus, we wanted to know if the other clinical parameters interfered in disease activity. The findings showed that neither prior use of methotrexate nor presence or absence of comorbidities interfered in disease activity, *p* = 4823 and *p* = 1182, respectively (Data not shown).  Table 1 details the clinical features of patients.

### 3.2. IL17A, IFNG, and FOXP3 mRNA Levels Were Increased in Skin Biopsy from Moderate-Severe Psoriatic Patients

**Figure 1:**
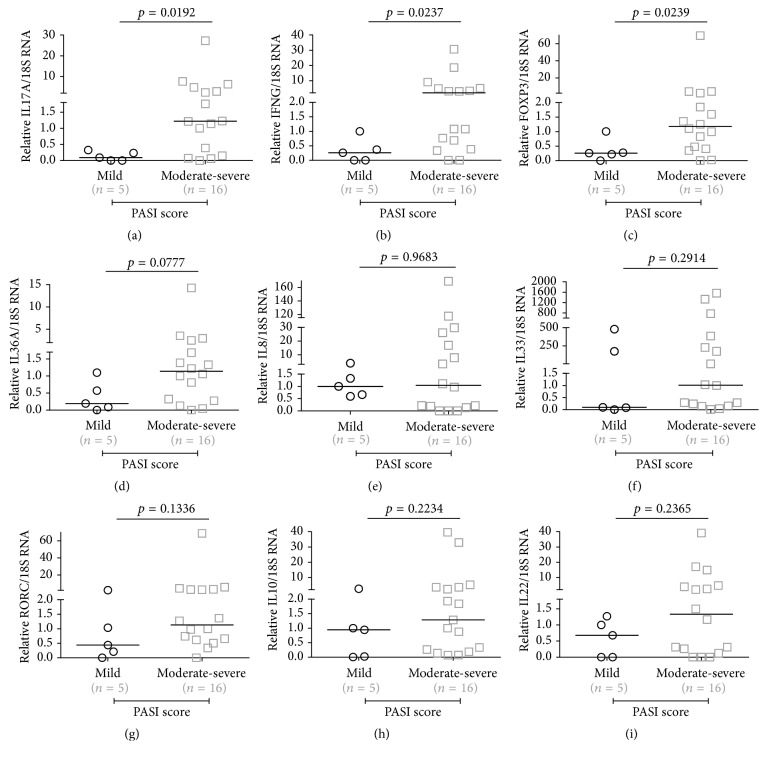
Transcripts levels of (a) IL17A, (b) IFNG, (c) FOXP3, (d) IL36A, (e) IL8, (f) IL33, (g) RORC, (h) IL10, and (i) IL22 according to PASI's severity disease.

We observed that in our sample only five individuals showed disease activity considered mild and the others had more severe clinical presentation. As we can see in Figures 1(a), 1(b), and 1(c), only three of the nine transcripts analysed showed statistical significance in moderate-severe disease compared with mild activity. IL17A, IFNG, and FOXP3 showed *p* = 0.0192, *p* = 0.0237, and *p* = 0.0239, respectively. Figures 1(d), 1(e), 1(f), 1(g), 1(h), and 1(i) provide the other analysed transcripts graphs.

### 3.3. Correlation between Cytokines in Skin Biopsies from Psoriatic Patients

After analysing the PASI score and their influence in expression of a panel of both pro- and anti-inflammatory transcripts, we evaluated the importance that each cytokine could play over the expression of the others. We summarized the significances and correlation's coefficients in Table 2.

In our study, we identified several statistically significant positive correlations between the molecules analysed in different magnitudes. With *p* < 0.05, IL17A correlated with its own transcription factor, RAR-related orphan receptor C (RORC), and other cytokines, such as IL22 and IL8. We also correlated IFNG versus IL10 and FOXP3; IL22 versus IL8 and IL10; RORC versus IL10; and lastly IL8 versus IL10.

We observed more significant correlation (*p* < 0.01) between IL36A versus IL17A, IFNG, IL8, and IL10. Similarly, FOXP3 showed correlation with IL10 and IL17A with IFNG. Strongly significant correlation (*p* < 0.001) was observed between the two transcription factors RORC versus FOXP3. This last one also correlated with IL36A and IL17A. Finally, IL10 showed significant correlation with IL17A.

### 3.4. Men and Women Have Different Profile of Cytokines Expressed in the Skin

**Figure 2:**
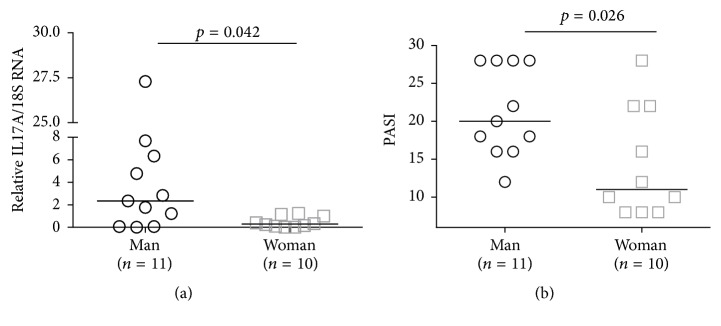
Different IL17A biopsy profile expression (a) and disease severity (b) between man and woman.

**Figure 3:**
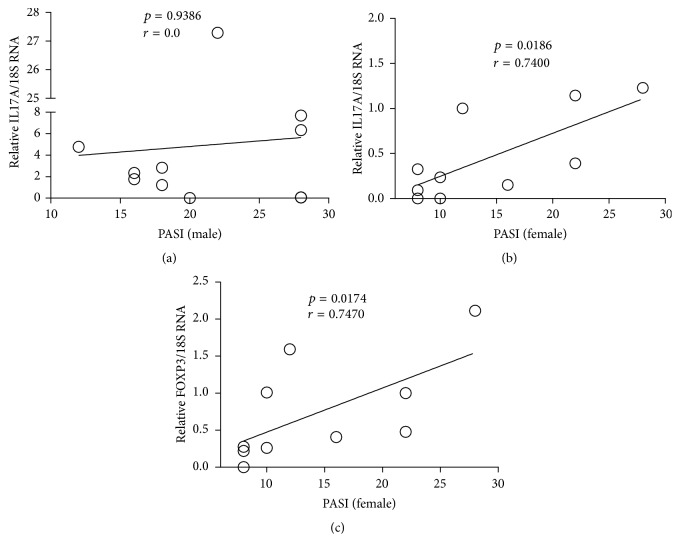
Correlation of IL17A (a, b) and FOXP3 (c) with disease severity between man and woman.

As previously mentioned, men and women exhibited different gravity of disease. Therefore, we investigated whether this fact influences the cytokine expression in the skin, separating the groups by gender. We observed that the male group showed significant high levels of IL17A transcripts compared to woman as well as more severe disease (Figures 2(a) and 2(b)). However, there was no significant statistical when we correlated each other (Figure 3(a)). In contrast, it occurred in female group. IL17A and FOXP3 transcripts showed significant statistical positive correlation with PASI (Figures 3(b) and 3(c)).

Among the fifteen statistically significant correlations described above, eight correlations only remained in the male group. They were IL17A versus, IL8, IL22, IL36A, and IL10, IL10 versus IL8 and IL22, IL36A with IL8, and RORC versus FOXP3. In the opposite, three correlations occurred only in female group: IL17A versus IFNG and FOXP3 and this last one with IL36A (Table 3).

### 3.5. IL17A Exhibits Greater Influence on Disease Severity

Finally, in order to understand the relationship between transcripts levels and PASI score, we conducted a multiple logistic regression. We found that IL17A high levels increased the chance to have moderate-severe disease as shown in Table 4. Due to the multicollinearity existence, IFNG and IL10 could not be included in the regression model.

## 4. Discussion

Recently, we demonstrated that IL-17A, IL-22, and IL-6 cytokines were more elevated in serum from psoriasis patients than in heathy controls. However, we have not found any correlation between systemic cytokine expression and disease severity [19]. So, we decided to investigate the lesion microenvironment and investigate if there was a correlation with disease severity and if it occurred in a local level in our group.

Over the past years, researches focused in Th1/Th17 pathways and described high levels of IFN*γ*, IL-17, and its isoforms in lesional psoriatic skin in both gene and protein levels [20–23]. Despite the availability of studies of psoriasis large-scale genomic and transcriptomes platforms [24–26], there is still a gap between the current knowledge and the clinical progression.

Our study showed increased expression of IL17A, IFNG, and FOXP3 in patients who exhibited severe clinical disease profile. Just as we did, Kim and Colleagues (2016) found significant difference in IL17A transcript expression between their clinically stratified groups [27]. In contrast to our study, they showed that patients classified with mild disease activity had the highest levels of IL17A. Suárez-Fariñas et al. (2012) also detected greater expression of IL23 p19 and p40 subunits IL17, IL22, and IFNG in lesions of moderate-severe patients using real-time reverse transcriptase method [28].

Further, we verified significant levels of FOXP3 in psoriasis lesions from severe patients. Considering that it is reported as a T regulatory cell transcription factor, we did not expect this increase. Soler and McCormick (2011) demonstrated that psoriasis patients really had regulatory T cells presenting FOXP3, although most of them were nonfunctional [29].

We did not observe significant differences in expression of IL8, IL22, IL10, RORC, IL33, and IL36A. However, we detected their signature in psoriatic skin at a transcript level and this corroborated with previous studies[9, 30, 31].

Continuing our analysis, we conducted all possible correlations between cytokines. Then, we found statistically significant correlation between cytokines from all three pathways with each other. It is important to notice that IL17A showed correlation with almost all analysed transcripts. Carrier and colleagues (2011) previously identified the interregulation among Th17 cytokines and IL-36 homologous forms. They also verified significance in correlation between IL36A versus IL17A and IFNG [32], as we did.

Curiously, we noted that IL17A had strong significant correlation with anti-inflammatory FOXP3 and IL10. We did not expect increased FOXP3 transcripts in moderate-severe patients, since this correlation seems antagonistic. Nevertheless, recent studies demonstrated another side of Treg cells. Bovenschen et al. (2011) found positive triple CD4^+^ IL-17^+^ FOXP3^+^ in lesions from psoriasis patients indicating copathogenic profile. Soler and colleagues (2013) also pointed the connection between IL17 and FOXP3. They defended that Treg cells in psoriasis readily turn into IL-17-expressing cells [33, 34].

We also verified that when we considered gender, some correlations between cytokines only occurred in one of the groups. The male group preserved most of correlations especially those involving Th17 pathway. We could associate it to the fact that male group showed PASI mean 21.3 ± 5.8 and it is statistically higher than in women PASI 14.4 ± 7.2. It is worth pointing out that the severity of psoriasis in female patients may fluctuate with hormonal changes with worsening in puberty and peak at menopause [35]. Taking into consideration that population in this study consisted of eldered women (57.7 ± 9.8), they presented milder disease.

It is important to recognize our limitations in this study. We identified some failures such as small sample size, elevated cases of patients with moderate-severe disease, absence of healthy controls, and lack of proteomic analysis to confirm our findings. However, we know that, in psoriasis research, there are few studies with emphasis on experimental immunology in Brazil. Therefore, we hope that these data may open doors for future investigations.

## 5. Conclusion

Our study revealed increased transcripts levels of IL17A, IFNG, and FOXP3 in moderate-severe psoriasis patients. IL17A seems to play major influence in disease severity. In addition, we also showed complex network correlations between Th1, Th17, Treg, and IL-1 family related transcripts.

## Figures and Tables

**Table 1:** Clinical features of psoriatic patients from Brazil northeast^a^.

Characteristics	All individuals (*n* = 21)
Age (yrs.)	
Mean ± SD/range	52.05 ± 13.48/23–74
Gender* N *(%)	
Male	11 (52.3)
Female	10 (47.7)
Disease duration (years)	
Mean ± SD (range), all	8.1 ± 6.5 (0.5–22)
0–5 years *N* (%)—mean ± SD	9 (42.8)—2.1 ± 1.2
6–10 years *N* (%)—mean ± SD	6 (28.6)—9.1 ± 1.3
>10 years *N* (%)—mean ± SD	6 (28.6)—16.3 ± 4.2
PASI clinical subgroups* N *(%)	
Mild (PASI < 10)—mean ± SD	5 (23.8)—8.8 ± 1
Moderate-severe (PASI ≥ 10)—mean ± SD	16 (76.2)—20.8 ± 5.7
Clinical comorbidities* N *(%)	
Diabetes	3 (14.3)
Dyslipidemia	3 (14.3)
Hypertension	3 (14.3)
Treatment* N *(%)	
Methotrexate	9 (42.9)

^a^Considering a Gaussian distribution, clinical values were represented by mean ± SD.

**Table 2:** Interplay among cytokines in human psoriasis lesions^a^.

	IL8	IL-33	IL36A	IL17A	FOXP3	RORC	IL22	IFNG	IL10
IL8		*p* = 0.443	*p* = 0.006	**p** = 0.037^**∗**^	*p* = 0.064	*p* = 0.798	**p** = 0.044^**∗**^	*p* = 0.087	**p** = 0.039^**∗**^
		*r* = −0.176	*r* = 0.574	**r** = 0.457	*r* = 0.410	*r* = 0.059	**r** = 0.442	*r* = 0.382	**r** = 0.452
IL33	*p* = 0.443		*p* = 0.106	*p* = 0.126	*p* = 0.097	*p* = 0.278	*p* = 0.857	*p* = 0.279	*p* = 0.211
	*r* = −0.176		*r* = 0.362	*r* = 0.344	*r* = 0.371	*r* = 0.248	*r* = −0.041	*r* = 0.247	*r* = 0.284
IL36A	**p** = 0.006^**∗∗**^	*p* = 0.106		**p** = 0.001^**∗∗**^	**p** < 0.001^**∗∗∗**^	*p* = 0.096	*p* = 0.117	**p** = 0.007^**∗∗**^	**p** = 0.002^**∗∗**^
	**r** = 0.574	*r* = 0.362		**r** = 0.651	**r** = 0.774	*r* = 0.372	*r* = 0.351	**r** = 0.563	**r** = 0.622
IL17A	*p* = 0.037	*p* = 0.126	*p* = 0.001		**p** < 0.001^**∗∗∗**^	**p** = 0.017^**∗**^	**p** = 0.049^**∗**^	**p** = 0.004^**∗∗**^	**p** < 0.001^**∗∗∗**^
	*r* = 0.457	*r* = 0.344	*r* = 0.651		**r** = 0.697	**r** = 0.511	**r** = 0.434	**r** = 0.598	**r** = 0.795
FOXP3	*p* = 0.064	*p* = 0.097	**p** < 0.001^**∗∗∗**^	**p** < 0.001^**∗∗∗**^		**p** < 0.001^**∗∗∗**^	*p* = 0.109	**p** = 0.020^**∗**^	**p** = 0.006^**∗∗**^
	*r* = 0.410	*r* = 0.371	**r** = 0.774	**r** = 0.697		**r** = 0.686	*r* = 0.359	**r** = 0.500	**r** = 0.574
RORC	*p* = 0.798	*p* = 0.278	*p* = 0.096	**p** = 0.017^**∗**^	**p** < 0.001^**∗∗∗**^		*p* = 0.821	*p* = 0.415	**p** = 0.049^**∗**^
	*r* = 0.059	*r* = 0.248	*r* = 0.372	**r** = 0.511	**r** = 0.686		*r* = 0.052	*r* = 0.187	**r** = 0.433
IL22	**p** = 0.044	*p* = 0.857	*p* = 0.117	**p** = 0.049^**∗**^	*p* = 0.109	*p* = 0.821		*p* = 0.179	**p** = 0.024^**∗**^
	**r** = 0.442	*r* = −0.041	*r* = 0.351	**r** = 0.434	*r* = 0.359	*r* = 0.052		*r* = 0.304	**r** = 0.487
IFNG	*p* = 0.087	*p* = 0.279	**p** = 0.007^**∗∗**^	**p** = 0.004^**∗∗**^	**p** = 0.020^**∗**^	*p* = 0.415	*p* = 0.179		**p** = 0.028^**∗**^
	*r* = 0.382	*r* = 0.247	**r** = 0.563	**r** = 0.598	**r** = 0.500	*r* = 0.187	*r* = 0.304		**r** = 0.476
IL10	**p** = 0.039^**∗**^	*p* = 0.211	**p** = 0.002^**∗∗**^	**p** < 0.001^**∗∗∗**^	**p** = 0.006^**∗∗**^	**p** = 0.049^**∗**^	**p** = 0.024^**∗**^	**p** = 0.028^**∗**^	
	**r** = 0.452	*r* = 0.284	**r** = 0.622	**r** = 0.795	**r** = 0.574	**r** = 0.433	**r** = 0.487	**r** = 0.476	

^a^Determination of statistical correlations was made according to Spearman's rank correlation test and represented by *p* value. **∗** equals *p* < 0.05, **∗** **∗** *p* < 0.01, and **∗** **∗** **∗** *p* < 0.001. The correlation coefficients are represented by “*r*.”

**Table 3:** Correlation coefficients and *p* values by gender^a^.

Gene	Man	Gene	Woman
IL17A vs IL8	*p* = 0.017	IL17A vs IFNG	*p* < 0.001
	*r* = 0.716		*r* = 0.976
IL17A vs IL22	*p* = 0.041	IL17A vs FOXP3	*p* = 0.049
	*r* = 0.633		*r* = 0.644
IL17A vs IL36A	*p* = 0.031	FOXP3 vs IL36A	*p* < 0.001
	*r* = 0.664		*r* = 0.806
IL17A vs IL10	*p* = 0.001	—	—
	*r* = 0.882		
IL10 vs IL8	*p* = 0.042	—	—
	*r* = 0.633		
IL10 vs IL22	*p* = 0.034	—	—
	*r* = 0.651		
IL36A vs IL8	*p* = 0.024	—	—
	*r* = 0.688		
RORC vs FOXP3	*p* = 0.005	—	—
	*r* = 0.800		

^a^Determination of statistical correlations was made according to Spearman's rank correlation test and represented by *p* value. *p* < 0.05, *p* < 0.01, and *p* < 0.001. The correlation coefficient is represented by “*r*.” “vs” means versus.

**Table 4:** Association of transcripts expression and disease activity^a^.

PASI score	Odds ratio	95% CI^+^	*p* value
IL22	0.189	0.005–6.247	0.351
RORC	0.388	0.053–2.825	0.350
FOXP3	0.014	6.18e − 07–344.728	0.411
*IL17A*	8.16e − 06	5.49e − 10–0.121	*0.017*
IL36A	21.499	0.191–2414.25	0.203
IL33	1.000	0.989–1.012	0.881
IL8	1.686	0.984–2.888	0.057

^a^CI^+^: confidence interval.
